# Spatial stereoresolution for depth corrugations may be set in primary visual cortex

**DOI:** 10.1186/1471-2202-12-S1-P263

**Published:** 2011-07-18

**Authors:** Fredrik Allenmark, Jenny Read

**Affiliations:** 1 Institute of Neuroscience, Newcastle University, Newcastle upon Tyne, UK

## 

Stereo depth perception has recently been modelled based on local cross-correlation between the left and the right eye’s images. This model, which is based on the known physiology of primary visual cortex (V1), has successfully explained many aspects of stereo vision. In particular, it has explained the low spatial stereoresolution for sinusoidal depth corrugations [1,2], suggesting that the limit on stereoresolution may be set in V1. In accordance with the properties of V1 neurons, the disparity detectors used in this model are tuned to locally uniform patches of disparity. Consequently, the model responds better to high amplitude square-wave corrugations than to high amplitude sine-waves, because the square-waves are locally flat while the sinusoidal corrugations are slanted almost everywhere and this slant is particularly large at large amplitudes. The model therefore predicts better performance at detecting square-wave than sine-wave disparity corrugations at high amplitudes. However, in contradiction with this prediction of the model we have recently shown that humans perform no better at detecting square-waves than sine-waves even at high amplitudes [3]. This failure of the model raised the question of whether stereoresolution is not set in V1 but at some later stage of cortical processing, for example involving neurons tuned to slant or curvature or whether a modified version of the model, incorporating more of the known physiology, may explain the new results with square-waves. We have tested a modified version of the local cross-correlation model which, based on psychophysical and physiological evidence that larger disparities are detected by neurons with larger receptive fields (a size-disparity correlation), uses larger windows to detect larger disparities. We show that the performance of this modified model is consistent with the human results, confirming that stereoresolution may indeed be limited by V1 receptive field sizes.
